# Homogeneity and Best Practice Analyses in Hospital Performance Management: An Analytical Framework

**DOI:** 10.1007/s10729-022-09590-8

**Published:** 2022-02-22

**Authors:** Mansour Zarrin, Jan Schoenfelder, Jens O. Brunner

**Affiliations:** Department of Health Care Operations / Health Information Management, Faculty of Business and Economics, University of Augsburg, Universitätsstraße 16, 86159 Augsburg, Germany

**Keywords:** Cluster Analysis, Data Envelopment Analysis, Hospital Efficiency Analysis, Artificial Neural Networks, Heterogeneity Analysis

## Abstract

Performance modeling of hospitals using data envelopment analysis (DEA) has received steadily increasing attention in the literature. As part of the traditional DEA framework, hospitals are generally assumed to be functionally similar and therefore homogenous. Accordingly, any identified inefficiency is supposedly due to the inefficient use of inputs to produce outputs. However, the disparities in DEA efficiency scores may be a result of the inherent heterogeneity of hospitals. Additionally, traditional DEA models lack predictive capabilities despite having been frequently used as a benchmarking tool in the literature. To address these concerns, this study proposes a framework for analyzing hospital performance by combining two complementary modeling approaches. Specifically, we employ a self-organizing map artificial neural network (SOM-ANN) to conduct a cluster analysis and a multilayer perceptron ANN (MLP-ANN) to perform a heterogeneity analysis and a best practice analysis. The applicability of the integrated framework is empirically shown by an implementation to a large dataset containing more than 1,100 hospitals in Germany. The framework enables a decision-maker not only to predict the best performance but also to explore whether the differences in relative efficiency scores are ascribable to the heterogeneity of hospitals.

## Highlights

- A novel framework for homogeneity and best practice analyses of hospitals
- Combining DEA with artificial neural networks for clustering and homogeneity analysis
- Study the influence of heterogeneity of hospitals on the relative efficiency
- Best performance predictions that reveal a large potential for improvement
- Supporting managers in designing a stepwise efficiency improvement plan

## Introduction

The Federal Statistical Office^1^ of Germany reports that the costs of inpatient hospital care amounted to around 91.3 billion euros in 2017, 3.9% higher than in 2016 (87.8 billion euros). Health care costs are driven primarily by hospitals around the world. Because of this, hospitals must constantly monitor and improve their efficiency. Data Envelopment Analysis (DEA) is one of the most effective tools for measuring efficiency, and it is widely used to evaluate the efficiency of decision-making units (DMUs). Nowadays, the use of DEA is rapidly expanding and its usage for hospital efficiency measurement is widely accepted (Kohl et al. 2019). In particular, basic DEA models have two major issues including restrictions by some fundamental assumptions such as homogeneity of DMUs in the dataset (Dyson et al. 2001; Brown 2006) as well as lack of predictive capabilities while they are frequently used as a benchmarking tool. In the following, we introduce these two issues and then explain the main aims of our study.

### Homogeneity

In the DEA context, homogeneity of a set of DMUs means that all DMUs operate in the same environment and pursue the same target with the same processes. Although significant research has been conducted on the heterogeneity of DMUs, many studies utilize the homogeneity assumption as pointed out by Haas and Murphy (2003) and Wojcik et al. (2019). The applicability of the homogeneity assumption in a sample is usually based on the implicit knowledge of investigators conducting DEA (Dyson et al. 2001). As elucidated by Samoilenko and Osei-Bryson (2010), two factors are important to assume the homogeneity of DMUs in DEA models. The first one that is known as semantic homogeneity brings up the common sense and logic concerned with the meaning assigned to all DMUs in the sample by decision-makers. The second factor is scale homogeneity, where the decision-maker must ensure that the functional similarity of DMUs would not be affected by the input and output levels. Paying no attention to either of these assumptions can heavily influence the results of a DEA application (Dyson et al. 2001). The differences may stem from the type of ownership, the hospital size, and the differences in political and legal environments where the hospitals operate. In the production process, environmental variables are not considered to be traditional inputs and are assumed to be out of the managers’ control. The debate about the best ways to incorporate these variables into DEA is still ongoing. Even assuming that the complete consideration of all influential environmental variables is possible, this will cause a lower level of discrimination because of the resulting substantial increase in the number of inputs and outputs (Dyson et al. 2001; Samoilenko and Osei-Bryson 2010).

The impact of the hospital environment can be modeled implicitly by grouping similar DMUs to their transformation capacity (or technology) together. This requires a technique that uncovers categories in the large and multidimensional dataset of DMUs. Incorporating environmental variables in DEA studies has traditionally relied on the two-stage model (Cooper et al. 2011). This approach employs the traditional inputs and outputs in the first stage to compute DEA efficiency scores, which are then regressed against the environmental variables (Simar and Wilson 2007). Since both ends of the $0-1$ distribution are restricted, it is often appropriate to use a censored regression model (such as Tobit) for these data. DEA estimates are corrected for environmental effects using regression coefficients. As a result, all efficiency scores will be aligned with the same environment, say the sample mean. However, there is a flaw in this approach. In classical regression, variables are assumed to be independent and identically distributed. According to Simar and Wilson (2007), the DEA efficiency scores considered as the dependent variable in the regression analysis are serially correlated. Therefore, conclusions from the results of this type of study should be drawn with caution. Rather, the method can be regarded as exploratory, indicating which environmental variables are most influential in performance. Another acknowledged approach (Brown 2006; Dyson et al. 2001) to address this issue is to cluster the DMUs into homogenous sets according to some similarities in their environment. Using cluster analysis, we can identify homogeneity between different clusters based on their similarity.

**Fig. 1:**
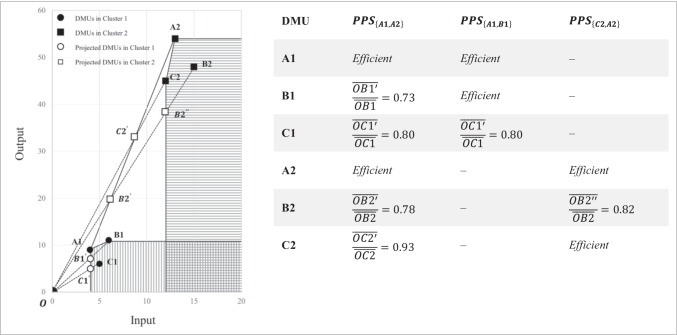
Contribution of clustering to measuring efficiency

To illustrate how clustering may improve efficiency estimates, consider a sample of 6 DMUs that use an input to generate one output, as shown in Figure 1. DEA benchmarks actual DMU behavior against a set of best practice frontiers. These frontiers create the production possibility set (PPS). As a measure of overall performance, the distance from the DMUs to the frontier is calculated. Best practices, therefore, play a prominent role in calculating the efficiency score. Figure 1 below shows the differences between three different PPSs. As we perform a DEA to measure the efficiency of all six DMUs together, DMUs $A1$ and $A2$ create the efficient frontier. The PPS consists of the area enclosed by this efficient frontier line, plus the horizontal line that extends down from $A1$ and the vertical line that extends right from $A2$. The four DMUs $B1$, $C1$, $B2$, and $C2$ are identified by the DEA as inefficient, and their efficiency can be evaluated by referring to the frontier lines. The efficiency of $B1$, for example, within this PPS is evaluated by $\overline{OB{1 }^{^{\prime}}}/\overline{OB1 }=0.73$. This unit is inefficient since it underperforms compared to the set of efficient DMUs: $\left\{A1, A2\right\}$. It is referred to as the *reference set* or *peer group* of the DMU $B1$. Nevertheless, when we implement clustering before running the DEA, two distinct clusters are detected: cluster 1 (vertical stripes area) includes $A1$, $B1$, and $C1$, and cluster 2 (horizontal stripes area) includes $A2$, $B2$, and $C2$. In cluster 1, the efficient frontier is formed by $A1$ and $B1$, the DMU that was previously shown to be inefficient. $C2$, the DMU that was previously indicated as inefficient, now forms the efficient frontier of cluster 2 together with $A2$. This example illustrates how the clustering can contribute to the estimation of efficiency behind identifying similar DMUs forming the PPS. Clustering may be a useful approach for determining homogeneity and heterogeneity in data sets. To help identify homogenous groups, clustering techniques maximize homogeneity within a group and heterogeneity between groups. Therefore, the resulting inefficiency scores will not be influenced by, e.g., economies of scale.

Traditional DEA models can present several traps for the unwary because of the issue of homogeneity. By analyzing the transformative capacity of hospitals, this study aims to examine the source of differences in the inefficiency of hospitals.

### Predictive capabilities

When managers of inefficient hospitals receive the results of a DEA, they usually have subsequent requests, including the possibility of keeping a watchful eye on progress by analyzing what-if scenarios during operational phases and setting target performance levels. Therefore, hospitals must be capable of setting up actionable targets that are specific and measurable. Additionally, analyzing hypothetical scenarios via an adaptive estimation capability can be a valuable addition to assist managers in the monitoring process during the operational phase of change. Although there have been successful models to measure the comparative efficiency of competing units, little attention has been given to including predictability in the performance measurement framework (Kohl et al. 2019). As a second objective, this study explores what level of improvement is needed to see an inefficient hospital become efficient by approximating the efficient frontiers of each cluster and predicting the best performance of each inefficient hospital within its cluster (compared to its leader). Additionally, it facilitates the controlling process during implementation by adding value to if-then scenarios.

## Literature Review and Contribution

This section reviews the literature relevant to DEA models, neural networks in DEA, clustering in DEA, and the hypothesis tests developed for comparing two groups of DMUs. This section also summarizes our contribution to the literature.

### Model Selection

The basic DEA model introduced by Charnes, Cooper, and Rhodes, known as CCR, evaluates the relative efficiency of a set of DMUs (Charnes et al. 1978). Using a variable return-to-scale (VRS) setting, Banker et al. (1984) advance the CCR model. This model is called the BCC model. As radial models, CCR and BCC deal with proportional changes in outputs or inputs. Using these models, the efficiency score is the proportional maximum output (or input) expansion (or reduction) ratio common to all outputs (or inputs) (Tone 2017, 2001). The assumption that these factors will behave proportionally is too restrictive in real-world situations. A further limitation of radial models is ignoring slacks in calculating efficiency scores. Non-radial Slacks-Based Measure (SBM) models have been developed to address these restrictions. SBM DEA models do away with the proportional change assumption and deal directly with slacks. The DEA model has been recognized to be a powerful tool for performance analysis and benchmarking, spanning a wide range of industries and functional areas, including healthcare (Kohl et al. 2019; Almeida Botega et al. 2020; Araújo et al. 2014). In a recent study on the German hospital market, Schneider et al. (2020) investigate hospital urgency scores (noting the average level of medical urgency in all cases treated at a hospital) are compared to technical efficiency. They use the data of 1,428 hospitals throughout Germany for the years 2015, 2016, and 2017. Simar and Wilson (1998) promote bootstrapping as a resampling method for DEA, which has become one of the most commonly used methods in hospital DEA applications (Kohl et al. 2019). There are two main reasons why it is relevant to DEA. DEA estimates tend to be positively biased (Nedelea and Fannin 2013; Mitropoulos et al. 2014) because the estimated production frontier is determined by the units included in the sample. A DMU does not use every input/output combination that is theoretically possible. Hence, the estimated frontier of efficient DMUs is typically too low, even if efficient DMUs are not missing for other reasons (Simar and Wilson 2004). DEA, therefore, assigns efficiency scores that are biased upward because the DMUs are assumed to be closer to the production frontier than they actually are. This upward bias can be corrected via the bootstrapping procedure by creating significance intervals for the efficiency estimates. Our study uses an input-oriented SBM DEA model, in contrast to previous studies (Kwon 2017; Samoilenko and Osei-Bryson 2010, 2008; Omrani et al. 2018), which mostly utilized radial models. We conduct a statistical analysis to determine whether the SBM estimates are significantly biased upward in comparison to the bootstrapped DEA model.

### DEA and Machine Learning

Few studies have attempted to reinforce DEA models with machine learning such as artificial neural networks (ANNs) for hospital performance evaluation despite the established effectiveness of these approaches (Kohl et al. 2019). Generally, incorporating ANNs with DEA can be categorized into two distinct research streams. The first consists of studies comparing DEA to ANN as an alternative way of assessing efficiency (Athanassopoulos and Curram 1996; Santín et al. 2004). According to the second stream of research, ANN can be used as a complement to DEA to gain potential advantages. Clustering is one of the machine learning methods used in the literature for subdividing a dataset of DMUs into subsets (clusters) according to how similar the observations are within each cluster. Several algorithms have been developed in the literature for conducting clustering (Saxena et al. 2017). Among these techniques, three general approaches comprising hierarchical, two-step, and partitional clustering have been used as complements to DEA to handle the scale heterogeneity of samples in the dataset (Mahmoudi et al. 2019; Omrani et al. 2018; Samoilenko and Osei-Bryson 2010). The application of clustering in the literature can be divided into two approaches. One approach is applying clustering to the results of a DEA to facilitate creating multiple reference subdivisions from the original set of DMUs (Bojnec and Latruffe 2008). Second, each DMU is compared with only a subset of its reference set. In the presence of dataset heterogeneity, we can use this approach to isolate the multiple homogenous subsets (Herrera-Restrepo et al. 2016; Samoilenko and Osei-Bryson 2010). In clustering, it is also important to specify the appropriate number of clusters. The quality of partition and cluster validity has been assessed by several authors using different indices (Rocci and Vichi 2008). The Caliński-Harabasz index (CH-index), the Silhouettes, and the Davies-Bouldin criteria were found to be acceptable in a study of clustering conducted by Łukasik et al. (2016). In the literature, details regarding these two criteria and how they are calculated can be found, for example, in Ünlü and Xanthopoulos (2019).

### Efficiency Comparison

This study advances the benchmarking paradigm suggested by Samoilenko and Osei-Bryson (2010), which is an extension of Samoilenko and Osei-Bryson (2008), by successfully integrating the clustering and ANN prediction models into an SBM DEA. In Samoilenko and Osei-Bryson (2010), the averages of the relative efficiencies of clusters are used to analyze heterogeneity. A cluster that has a higher average efficiency is referred to as a leader, and a cluster with a lower average efficiency is referred to as a follower. Their method is imprecise because they compare DEA estimates using the mean value of the efficiency scores without considering the distribution of the estimates. The mean value becomes an inappropriate measure when the frequency distribution of the efficiency scores is skewed (Weisberg 1992). Several studies have been conducted where DEA estimation distributions between two groups of DMUs are compared by developing both parametric and non-parametric statistical tests. Banker et al. (2010) develop two sets of parametric and three non-parametric tests. The idea of comparing two groups of DMUs is combined with a heterogeneity analysis in our study. Additionally, we apply our framework to a setting with more than one pair consisting of one leader and one follower.

Our contribution proposes an analytical framework consisting of three stages. We design SOM-ANN for clustering, followed by an SBM DEA model that calculates the relative efficiency of the clustered hospitals. We develop two MLP-ANNs to generate: (i) the transformative capacity model (TCM) to analyze the homogeneity, and (ii) the best practice model (BPM) to predict the level of improvement desired, to achieve efficient operation. The rest of the paper follows this structure. In Section 3, we describe the research methodology and the multi-stage analytical framework combining SOM-ANN, SBM DEA, and MLP-ANN. The dataset of German hospitals used to demonstrate the framework’s applicability is presented in Section 4. The results of the implementation of the framework are presented in Section 5. Section 6 concludes with a discussion of future research directions and conclusions.

## Methodology

**Fig. 2:**
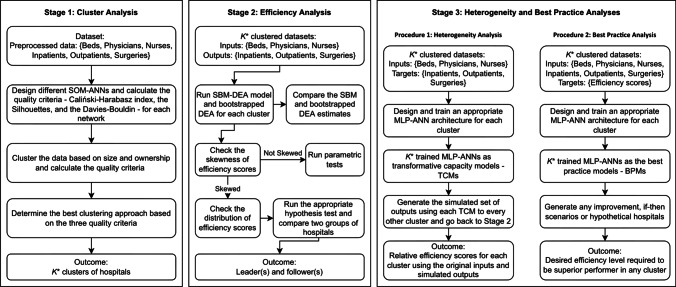
Proposed analytical framework

In this section, we describe our proposed framework (see Figure 2). The framework contains three main stages: 1) Clustering using SOM-ANN, 2) efficiency analysis, and 3) heterogeneity and predictability analyses. Each stage is described in detail in the following subsections.

### Stage 1: Cluster Analysis

We use an SOM-ANN architecture because SOMs are non-linear techniques that can summarize and analyze numerous aspects of variability in a complex, large, multivariate, multi-dimensional dataset (Hudson et al. 2011). In contrast to more traditional clustering methods (such as K-means), SOM-ANN, without imposing a structure on the input/output variables, identifies natural groupings by producing a succinct organization based on similarities among the transformation capacity. As network optimization remains a challenging task, SOM-ANN settings such as initial neighborhood size, topology, and distance functions have been determined by trial and error (Emrouznejad and Shale 2009; Kwon 2017). We also study alternative clustering approaches that are based on the hospitals’ natural characteristics: their size (number of beds) and ownership type. The size-related clusters are: small ($beds<500$), medium ($500\le beds<\mathrm{1,000}$), and large ($beds>\mathrm{1,000}$), while the ownership type clusters are: public, non-profit, and private. This allows us to determine whether natural clustering produces high-quality clusters for hospitals and, consequently, ensures homogeneity within those clusters by comparing the quality indicators calculated for SOM clustering and natural clustering. The function developed for our clustering approach in Python 3.8 is presented in Appendix A.

### Stage 2: Efficiency Analysis

We run the input-oriented SBM DEA model under VRS settings to calculate the efficiency score of each hospital in each cluster. The mathematical formulation is presented in Appendix B. We also provide details regarding how to calculate the projections based on the slacks determined by the SBM DEA model. In DEA applications, the orientation is chosen based on which parameters managers have more control over (Cooper et al. 2004). While marketers, referral sources, and other methods such as reputation management, can sometimes generate additional patients for hospitals (Ozcan 2014), we use an input orientation under the assumption that hospital managers can more readily control the resources used for patient treatments. Thus, we are interested in the amount by which the resources/inputs (e.g., staff) can be reduced proportionately without reducing the number of treated patients. The downside of using an input-oriented model is the limited applicability when demand for care is higher than the supplied capacity. While this situation may occur for specific treatment types such as chemotherapy or respiratory assistance temporarily, the German healthcare system is set up to continuously assess long-term capacity requirement projections and to react to demand changes with di-/investments into treatment capacities on a state level, so that supply and demand are balanced in the long run.

Furthermore, to determine whether SBM DEA estimates are biased upward or not, we perform a statistical test analysis (explained in the following subsection) between the SBM DEA estimates and bootstrapped DEA estimates produced by implementing the algorithm developed in Daraio and Simar (2007) with the conduct of 200 bootstrap iterations. For brevity, we will not repeat the steps of the algorithm here, however, the reader may refer to Daraio and Simar (2007) for more details.

#### Efficiency Comparison of Two Groups of Hospitals

A DEA estimator of the production frontier is a fully-fledged statistical methodology (Banker 1993) by which we can construct a variety of statistical tests based on efficiency scores represented as stochastic variables. Appendix C describes the comparison algorithm in detail. After indicating the existence of a statistical difference between ${G}_{1}$ and ${G}_{2}$, we reperform the appropriate tests under the one-tailed null hypothesis to indicate whether the efficiency of ${G}_{1}$ is greater than ${G}_{2}$ or vice versa. Throughout the study, all hypothesis tests are performed with a significance level of 5%. Following this procedure, we label the leader and follower in each pair of hospitals.

### Stage 3: Heterogeneity and Best Practice Analyses

In this stage, two MLP-ANN architectures are designed in two different ways, which are explained in detail in the following subsections. The first architecture supports the scale heterogeneity analysis and the second one is used to predict the actual output level necessary for an inefficient hospital to be efficient. The MLP-ANN maps complex unknown relationships in the dataset because (*i*) MLP-ANNs have a stochastic learning process, which minimizes the chance of being trapped in local minima, and (*ii*) there is no necessity to specify and know the relationships within the dataset. This architecture, the multilayer feedforward network, is mostly used with the backpropagation algorithm.

#### Heterogeneity Analysis

A model of transformative capacity for each cluster is generated by creating and training an MLP-ANN. Here, it is proposed that score estimates obtained from DEA can be indirectly employed to investigate the factors influencing relative efficiency scores (Hoff 2007; Samoilenko and Osei-Bryson 2010). The DEA efficiency score calculation, however, is hampered by the unavoidable misspecification of the model when determining which inputs are converted into which outputs. Therefore, the decision-maker needs to know the correct transformation function of inputs into the outputs used for conducting the modeling of these estimated scores by DEA. We generate and analyze the transformative capacity model for cluster $k$ denoted by $TC{M}_{k}$. For each cluster, the designed MLP-ANN is trained using the set of input variables (number of beds, physicians, and nurses) as input nodes and the set of output variables (number of adjusted inpatients, outpatients, and surgeries) as output nodes. This is analogous to the way that input data can be transformed into outputs by a given cluster. Then, we investigate for any leader-follower-pair whether the relative efficiency score of the follower improves when comparing the efficiency score distribution of the follower, using the simulated outputs of the follower employing $TC{M}_{k}$ of its leader $k$. When the efficiency score of the follower improves, there is a reason to recommend that the disparity between the original efficiencies of the leading and following clusters is partly due to the differences in transformative capacity. To analyze the scale heterogeneity (scalability), we use the original inputs and outputs of the follower and the initial inputs and simulated outputs of its leader obtained from the $TC{M}_{k}$ (follower $k$) for any leader-follower pairs. If the efficiency of the leader is still higher than the follower, then we can say that scale heterogeneity plays a part in explaining the disparity between the relative efficiencies of the leading and following cluster. In other words, even with the less efficient process of the transformative capacity (i.e., $TC{M}_{k}$, follower $k$), the leader remains relatively more effective. Visual description is given in Stage 3 of the framework presented in Figure 1 as “Procedure 1: Heterogeneity Analysis.”

#### Best Practice Analysis

The second MLP-ANN architecture is designed to deliver improved estimation precision due to its pattern mapping and learning capabilities as a complementary method to DEA. The objective of this analysis is to investigate the predictive capabilities of ANN when used alongside DEA. To this end, the MLP-ANN architecture is trained based on inputs and outputs of the hospitals in each cluster as the input layer and their SBM DEA efficiency scores (see Stage 2 in Figure 1) as the target nodes. Managers can benefit from this analysis in two different ways. First, in a capital-intensive and competitive environment such as in the hospital setting, the ability to estimate input/output levels beyond the calculated relative efficiency scores is essential for performance benchmarking in real-world applications (Ozcan 2014). Therefore, the first way this analysis can be used by decision-makers is to estimate the efficiency level that can be reached by using a given level of inputs to produce a given level of outputs. Second, the analysis allows managers to set stepwise improvement goals by utilizing what-if scenarios for each inefficient hospital to become an efficient unit, not only in its cluster but also in other clusters without requiring a new DEA. For example, we conduct further experiments to investigate the potential of the proposed framework based on the leader-follower strategy. While DEA has powerful optimization capabilities and a wide range of applications, it has restrictions when working with new or unobserved data sets. If a new DMU is added to a sample and the DEA model is rerun, the results might be completely different as this new DMU might alter the PPS. Hence, the second way this analysis helps managers is to calculate the relative efficiency score of a new or hypothetical hospital by using BPMs trained to learn efficiency patterns existing in the market. This provides managers with alternative paths leading toward best practices, which typically occur at the planning stage and before implementation. Visual description is also given in Stage 3 of the framework presented in Figure 1 as “Procedure 2: Best Practice Analysis.”

## Data Set and Descriptive Statistics

The proposed framework in this study is examined in the context of a large dataset of hospitals recorded by the Federal Joint Committee^2^ in Germany in 2017. The raw dataset includes all the hospital quality reports of the reporting year 2017. In this study, the information on standard input and output variables for performance assessment of hospitals (Kohl et al. 2019; Tone 2017) was extracted from these reports. Appendix D provides more details about the data sources and a flowchart of the steps involved in data preprocessing. The processed dataset includes 1,124 hospitals.

Kohl et al. (2019) provide some insights into standard input/output settings in their review of hospital DEA studies. Their report indicates that the parameters most used in hospital DEA applications are beds, nurses, physicians, inpatients, and outpatients. These measures are suitable for describing the service process of a hospital as stated by Ozcan (2014). A hospital’s capacity can be measured by the number of beds it has. Physicians and nurses play the main role in the hospital’s service process. Therefore, the input factors can be considered as beds (Beds), nurses (Nurses), and physicians (Physicians). In our sample, we use full-time equivalents (FTE) of physicians and nurses. As for the outputs, we use the most common output variables used in the literature (Kohl et al. 2019): the number of adjusted inpatients (Adjusted Inpatients) and the number of outpatients (Outpatients). Patients’ conditions need to be considered when evaluating inpatient cases, as not every patient requires the same level of care. Following a prior study on efficiency measuring of the German hospital market (Schneider et al. 2020), we apply the case-mix adjustment based on the relative length of stay for groups of hospital diagnoses (according to the International Classification of Diseases Tenth Revision [ICD-10] codes) as suggested by Herr (2008). The German Federal Statistical Office^3^ publishes hospital statistics on average lengths of stay for each diagnosis group. In addition to these outputs, we consider the number of surgeries based on OPS-5^4^ codes (Surgeries). This output plays a major role in generating net revenue for hospitals. Table 1 represents some descriptive statistics regarding the inputs and outputs of the hospitals in our dataset.

**Table 1:** Descriptive statistics of inputs and outputs of dataset (after preprocessing)

*Statistic*	*Beds*	*Physicians*	*Nurses*	*Adjusted Inpatients*	*Outpatients*	*Surgeries*
Mean	386.1	131.7	295.2	20,051.6	39,713.2	16,991.7
Standard Error	10.2	5.0	9.5	634.5	2,486.7	606.6
Median	283.0	79.7	199.6	12,262.1	20,780.0	9,795.5
StD	340.5	168.2	318.3	21,253.4	83,368.1	20,335.6
Kurtosis	9.8	28.3	20.7	15.6	137.6	11.9
Skewness	2.5	4.3	3.6	3.0	9.7	2.8
Minimum	50.0	6.0	11.0	628.8	11.0	1.0
Maximum	3,011.0	2,066.7	3,695.7	204,827.6	1,568,896.0	178,580.0
Sum	434,023.0	147,983.0	331,815.8	22,497,902.8	44,637,688.0	19,098,719.0
Confidence Level (95.0%)	19.9	9.8	18.6	1,244.9	4,879.0	1,190.1

^*^Including all types of physicians such as specialist, non-specialist, and external in full-time equivalent (FTE) unit.

^**^Including all types of nurses such as pediatric, geriatric, auxiliary, and general in the FTE unit.

## Results and Discussion

This section presents the key experimental results of each stage of the proposed framework. We interpret and explain how far these results support the hypothesis and answer the research questions.

### Results of Cluster Analysis

For the optimal number of clusters, we create a list of 54 distinct two-dimensional hexagonal layer topologies. We then run the SOM-ANN for each topology of this list to generate clustering vectors. For each clustering vector, three quality criteria are calculated: CH-index, Silhouettes, and Davies-Bouldin (see Appendix E). We then calculate the quality indicators for the clusters resulting from the size and ownership. The results are presented in Table 2. When compared to the best SOM clustering, size (small: $beds<500$, medium: $500\le beds<\mathrm{1,000}$, and large: $beds>\mathrm{1,000}$) and the ownership (public, non-profit, and private.) of hospitals provide low-quality clusters. Interestingly, clustering based on ownership is ineffective when identifying homogeneity within a group of hospitals and heterogeneity across groups, yet this approach is adopted often in DEA hospital applications with multiple stages (Ozcan 2014; Jacobs et al. 2006; Herr 2008). In identifying homogenous groups, size (number of beds) clustering performs better than ownership; however, they are both outperformed by SOM. By using SOM-ANN, we have three clusters and can calculate the efficiency scores of hospitals in each cluster.

**Table 2:** Results of comparing the clustering approaches

Clustering Approach	No. of hospitals			CH-index*	Silhouette**	Davies-Bouldin***
Size	Small: 853	Medium: 201	Large: 70	647.35	0.48	1.08
Ownership	Non-profit: 450	Private: 238	Public: 436	25.77	-0.11	7.59
SOM	Cluster 1: 186	Cluster 2: 249	Cluster 3: 689	874.54	0.57	0.76

^*^ A high score is achieved when clusters are dense and well separated.

^**^ The score ranges from $-1$ for incorrect clustering to $+1$ for dense and well-separated clustering.

^***^ A value closer to zero indicates a better partition.

### Results of Efficiency Analysis

We calculate the efficiency of each hospital and the projections calculated for each hospital using an input-oriented SBM DEA under the VRS setting. SBM DEA estimates (${G}_{SBM}$) are compared to bootstrapped DEA estimates (${G}_{BT}$) produced by the implementation of the algorithm developed by Daraio and Simar (2007) to determine if they are biased upward. Table 3 presents the results of the comparison. In all three clusters, efficiency scores are skewed. They follow neither an exponential nor a half-normal distribution.

**Table 3:** Comparison of bootstrapped DEA and SBM estimates

Cluster	Mean (Bootstrapped DEA, SBM)	StD (Bootstrapped DEA, SBM)	Median (Bootstrapped DEA, SBM)	*p*-value (${{\varvec{H}}}_{0}:\boldsymbol{ }{{\varvec{G}}}_{{\varvec{S}}{\varvec{B}}{\varvec{M}}}={{\varvec{G}}}_{{\varvec{B}}{\varvec{T}}};\boldsymbol{ }{{\varvec{H}}}_{1}:\boldsymbol{ }{{\varvec{G}}}_{{\varvec{S}}{\varvec{B}}{\varvec{M}}}\ne {{\varvec{G}}}_{{\varvec{B}}{\varvec{T}}}$)
1	(0.8078, 0.8300)	(0.1066, 0.1364)	(0.8259, 0.8465)	0.5540
2	(0.6439, 0.6862)	(0.1295, 0.1760)	(0.6469, 0.6575)	0.5650
3	(0.6797, 0.6891)	(0.1259, 0.1716)	(0.6808, 0.6610)	0.5332

Mann–Whitney tests reveal that the distribution underlying input-oriented SBM estimates is not significantly different from the distribution underlying bootstrapped DEA estimates. The *p*-values indicate that the null hypothesis should be retained. We then continue our analysis using the input-oriented SBM DEA model. Table 4 summarizes the results of the relative efficiency scores calculated for the clusters and all hospitals. As a result of clustering, both the mean and median efficiency scores as well as the number of efficient hospitals increase. Table 5 shows that the amounts by which inputs need to be reduced proportionately (while keeping the outputs constant) are significantly diminished after applying cluster analysis. For example, the number of beds that hospitals need to reduce, on average, to become efficient before clustering is 60% higher than after clustering. Clustering all hospitals in one group may conceivably distort the results since an important assumption of DEA is that all DMUs are homogenous.

**Table 4:** Descriptive statistics of efficiency scores before and after clustering

Statistics	Cluster 1		Cluster 2		Cluster 3	
	Before clustering	After clustering	Before clustering	After clustering	Before clustering	After clustering
Mean	0.7135	0.8300	0.6034	0.6862	0.5964	0.6891
Standard Error	0.0124	0.0100	0.0108	0.0112	0.0071	0.0065
Median	0.6898	0.8465	0.5905	0.6575	0.5633	0.6610
StD	0.1688	0.1364	0.1706	0.1760	0.1865	0.1716
Kurtosis	-0.1618	0.0765	0.4956	-0.5742	0.0077	-0.4841
Skewness	-0.0005	-0.5851	0.4991	0.3601	0.7116	0.3184
Minimum	0.2202	0.3352	0.2161	0.2973	0.1959	0.2516
Maximum	1.0	1.0	1.0	1.0	1.0	1.0
Efficient DMUs	20	39	9	34	41	84

**Table 5:** Descriptive statistics of input excesses before and after clustering

Statistics	Beds		Physicians		Nurses	
	Before clustering	After clustering	Before clustering	After clustering	Before clustering	After clustering
Mean	155.92	96.32	50.94	36.98	115.83	90.68
Standard Error	4.45	3.81	1.73	1.65	3.21	3.05
Median	120.25	57.30	34.66	20.42	85.34	63.91
Mode	0.00	0.00	0.00	0.00	0.00	0.00
StD	149.02	127.60	57.97	55.37	107.75	102.09
Kurtosis	28.48	48.18	15.45	21.59	12.93	17.23
Skewness	3.50	4.66	3.19	3.92	2.74	3.17
Maximum	2,062.56	1,982.96	568.81	559.61	1,169.14	1,165.43
Sum	175,254.98	108,266.58	57,253.24	41,565.68	130,189.20	101,929.65

### Results of Heterogeneity and Best Practice Analyses

This section presents the results of the last stage of the proposed framework. First, the simulated output sets for each cluster are generated based on the TCMs created by MLP-ANN. The first procedure of Stage 3 is focused on determining: (*i*) whether the relative efficiency score of hospitals in a certain cluster improves if we consider the TCM of other clusters, and (*ii*) identifying the differences that are partially due to scale heterogeneity. The second part of the analysis aims at exploiting the non-linear mapping capabilities of MLP-ANN by using the input and output data of each cluster as input nodes (input layer) and assigning their efficiency scores received from DEA-SBM as target nodes (output layer). We develop both MLP-ANNs using an end-to-end open-source platform called TensorFlow in Python 3.8. We set the mean absolute percentage error (MAPE) as the performance measure due to its scale independence, interpretability, and simplicity. For training, validation, and testing, we use a random data division function. The training function updates weight and bias values based on “Adam”, a stochastic optimization method developed by Kingma and Ba (2014). More details regarding the parameters of the developed MLP-ANNs are provided in Appendix F.

#### Results of Heterogeneity Analysis

For each cluster, we design an MLP-ANN to create a TCM (${TCM}_{k}, \forall k\in \left\{\mathrm{1,2},3\right\}$). Using the TCMs of the other two clusters, we simulate the output values of adjusted inpatients, outpatients, and surgeries for each cluster. For example, in the case of Cluster 1, we import the actual inputs (*Beds*, *Physician*, and *Nurses*) of this cluster to the TCMs generated for Cluster 2 (${TCM}_{2}$) and Cluster 3 (${TCM}_{3}$) to generate two simulated output sets for Cluster 1. The simulated outputs are then substituted for the actual outputs of Cluster 1, and the new relative efficiency scores are calculated. As a result, we have three sets of efficiency scores for Cluster 1 based on three sets of outputs: the original outputs, simulated outputs using TCM’s Cluster 2 (${TCM}_{2}$), and simulated outputs using TCM’s Cluster 3 (${TCM}_{3}$). ${C}_{k}^{TC{M}_{{k}^{^{\prime}}}}, \forall k,{k}^{^{\prime}}\in \left\{\mathrm{1,2},3\right\} and k\ne {k}^{^{\prime}}$ represents the set of relative efficiency scores calculated based on actual inputs of Cluster $k$ and the simulated outputs obtained from $TC{M}_{{k}^{^{\prime}}}$. The MAPE values calculated for each TCM are presented in Table 6. Surgeries show the highest MAPE value among the outputs, likely because its variance is higher than that of other outputs across all three clusters.

**Table 6:** Best settings of the designed MLP-ANNs for simulating outputs

Transformative capacity model	Layers	Train:Test:Validation Ratio	MAPE of the test dataset		
			Adjusted Inpatients	Outpatient	Surgeries
${TCM}_{1}$	[20, 10, 10]	75:20:5	15%	16%	24%
${TCM}_{2}$	[20, 10, 10]	80:15:5	7%	10%	14%
${TCM}_{3}$	[20, 10, 10]	80:15:5	6%	6%	11%

We must first define the leader-follower relationship for all cluster pairs by comparing the efficiency of two groups of hospitals. The efficiency scores of all clusters are skewed, as shown in Table 4. Following that, according to the algorithm developed for comparing efficiencies, we check whether the efficiency is distributed exponentially or half-normally for each pair of hospitals (${G}_{1}$ and ${G}_{2}$). Based on the Q-Q (Quantile-Quantile) plots of all clusters, they do not appear to have come from populations with an exponential or half-normal distribution. Therefore, we conduct the Mann–Whitney test to determine if one hospital cluster is stochastically more efficient than the other, i.e., determining the leader and the follower of the pair. Table 7 shows the results of comparing the distribution of efficiency scores of all clusters, including their leader and/or follower. There is no significant difference in efficiencies underlying Clusters 2 and 3. Therefore, in this pair, no leader (or follower) can be identified. However, if we only compare the mean efficiency scores (see Table 4) and determine the leader solely based on them, Cluster 3 emerges as the leader. In this regard, comparing the efficiency of two groups of hospitals only based on mean values could lead to the wrong detection of leaders. Based on the Q-Q plots of the simulated outputs, the new efficiency score sets are neither exponentially nor half-normally distributed. Therefore, we compare efficiency scores using the Mann–Whitney test (see Table 7).

**Table 7:** Comparing relative efficiency scores via Mann–Whitney test

*Pair* $\left\{{{\varvec{G}}}_{1},{{\varvec{G}}}_{2}\right\}$	*p-value* *(*${{\varvec{H}}}_{0}:\boldsymbol{ }{{\varvec{G}}}_{1}={{\varvec{G}}}_{2},{{\varvec{H}}}_{1}:\boldsymbol{ }{{\varvec{G}}}_{1}\ne {{\varvec{G}}}_{2}$*)*	*Result of hypothesis tests*	*Leader*
$\left\{{{\varvec{C}}}_{1},{{\varvec{C}}}_{2}\right\}$	0.0000	${C}_{1}>{C}_{2}$	${C}_{1}$
$\left\{{{\varvec{C}}}_{1},{{\varvec{C}}}_{3}\right\}$	0.0000	${C}_{1}>{C}_{3}$	${C}_{1}$
$\left\{{{\varvec{C}}}_{2},{{\varvec{C}}}_{3}\right\}$	0.6785	${C}_{2}={C}_{3}$	–

##### Transformative capacity

We utilize the actual inputs and the simulated outputs of the follower using $TCM$ of its leader and compare the resulting efficiency scores with the original efficiency of the follower. Consider the results reported in Table 8 for Clusters 1 and 2 as one instance. Cluster 1 is the leader of Cluster 2. The results indicate that the efficiency of Cluster 2 as a follower, based on its actual inputs and the ${TCM}_{1}$ outputs (${C}_{2}^{TC{M}_{1}}$), has increased compared with its initial efficiency score, i.e., ${C}_{2}<{C}_{2}^{TC{M}_{1}}$. This means that the difference between the relative efficiencies of Cluster 1 (leader) and Cluster 2 (follower) is caused by the disparities in their transformative capacities. However, this conclusion is not valid for Cluster 3 (${C}_{3}>{C}_{3}^{TC{M}_{1}}$) as the other follower of Cluster 1. For the pair $\left\{{C}_{2},{C}_{3}\right\}$, whose leader (follower) cannot be identified, this analysis should not be conducted. If we compared the mean values, the leader-follower analysis would proceed as follows: the average efficiency score ${C}_{2}^{{TCM}_{3}}$ is equal to 0.8838, a significant increase from the initial average efficiency score (0.6862). Thus, we could infer that the disparity in efficiency scores has to do with their differences in transformative capacity. However, as no leader/follower was identified in the first place, the efficiency distributions of the two clusters could not be determined to be significantly different. We can conclude that there are instances where the difference between the relative efficiencies of hospitals in Germany is due to disparities in their transformative capacities.

**Table 8:** Results of comparing relative efficiency scores calculated based on the TCMs via Mann–Whitney test

*Analysis*	*Leader*	*Follower*	${{\varvec{G}}}_{1}$	${{\varvec{G}}}_{2}$	*p*-value (${{\varvec{H}}}_{0}:\boldsymbol{ }{{\varvec{G}}}_{1}={{\varvec{G}}}_{2};{{\varvec{H}}}_{1}:\boldsymbol{ }{{\varvec{G}}}_{1}\ne {{\varvec{G}}}_{2}$)	*Result of hypothesis tests*
Transformative Capacity	1	2	${C}_{2}$	${C}_{2}^{TC{M}_{1}}$	0.0002	${C}_{2}<{C}_{2}^{TC{M}_{1}}$
	1	3	${C}_{3}$	${C}_{3}^{TC{M}_{1}}$	0.0000	${C}_{3}>{C}_{3}^{TC{M}_{1}}$
Scale Heterogeneity	1	2	${C}_{2}$	${C}_{1}^{TC{M}_{2}}$	0.0164	${C}_{2}<{C}_{1}^{TC{M}_{2}}$
	1	3	${C}_{3}$	${C}_{1}^{TC{M}_{3}}$	0.0000	${C}_{3}<{C}_{1}^{TC{M}_{3}}$

##### Scale heterogeneity (scalability)

We compare the original efficiency of a follower with the efficiency scores of its leader (based on the actual inputs and the simulated outputs by the TCM of the follower). Results are reported in Table 8. In the case of Clusters 1 and 2, the distributions of the initial efficiency score of the follower (${C}_{2}$) and the distributions of the efficiency score calculated based on ${TCM}_{2}$ for the leader (${C}_{1}^{TC{M}_{2}}$) are compared. Since ${C}_{1}^{TC{M}_{2}}$ is greater than ${C}_{2}$, Cluster 1 remains the leader of Cluster 2. Thus, scale heterogeneity partially explains the difference in relative efficiencies between Clusters 1 and 2. For Cluster 3, the other follower of Cluster 1, similar results can be observed (${C}_{3}<{C}_{1}^{TC{M}_{3}}$). Overall, there is no case in which the relative efficiency score of the leader is smaller than the relative efficiency score of the follower. There is no case in which the new relative efficiency scores of a leader are stochastically lower than those of the follower. In this way, we can argue that a part of the reason for the disparities between the relative efficiency scores of followers and leaders is scale heterogeneity. This indicates that in the German hospital market, despite the less efficient process of TCM (i.e., follower), the leading hospitals are relatively more efficient than the following ones.

#### Results of Best Practice Analysis

Similar to the first procedure, our next step is to find the best settings for the newly designed MLP-ANNs (i.e., BPMs) for our best practice analysis of hospitals. The performance measure of the trained BPMs is reported in Table 9. In each case, a low MAPE indicates a good fit and generalizability.

**Table 9:** Best settings of the designed MLP-ANNs for best practice analysis

*Cluster*	*Layers*	*Train*:*Test*:*Validation Ratio*	*MAPE of the test dataset*
1	[8, 8]	75:20:5	8%
2	[10, 10]	80:15:5	8%
3	[10, 10]	80:15:5	7%

The frontier function can be viewed as the upper limit of the support of the density of hospitals in the input and output space. On the efficient frontier, concavity and monotonicity assumptions are assumed to be preserved by DMUs. However, the bootstrapped estimates do not necessarily preserve the concave monotone increasing condition. As a result, BPMs are trained based on the SBM DEA estimates where concave monotonic properties of the efficient frontier are preserved (Pendharkar 2005, 2011; Kwon 2017).

To elaborate, we look at one inefficient hospital in Cluster 2, for instance, which has an efficiency score of 0.7422. The SBM DEA projections suggest reducing the number of beds by 27%, physicians by 21%, and nurses by 24%. In terms of output, the projection calls for increasing the number of outpatients by 16%, adjusted inpatients by 5%, and surgeries by 887%, which sounds unrealistic. It is now necessary for the management of this hospital to have a list of possible improvement scenarios that determine what efficiency level can be achieved by using a given level of inputs to provide a given level of outputs. Re-running the DEA for every scenario setting is one option. If, however, we want to keep the PPS unchanged, we cannot consider scenarios with lower reduction rates than those predicted by input projections or higher expansion rates than those set by output projections. By reducing beds by 35% and keeping the remaining factors unchanged, DEA might form a new PPS according to the new data. However, the designed BPM of Cluster 2 ($BP{M}_{2}$) can predict the desired level of this hospital’s best performance in any setting without concern over creating a new efficient frontier. Table 10 presents the estimation results on possible improvement scenarios for this hospital and shows the projected efficiency increase that can be achieved by decreasing inputs and/or increasing outputs. As we can see from Scenario 7, the management of the hospital under study does not have to follow the projections derived from the DEA (e.g., unrealistic increasing the number of surgeries by about 900%) to become efficient in the peer group. Compared to SBM projections, these changes sound more realistic and applicable. For varying input levels, the proposed approach can support managers in setting optimal levels of outputs (e.g., the number of adjusted inpatients or outpatients). The same analysis and investigation can be applied to every other inefficient hospital.

**Table 10:** Possible improvement scenarios for an inefficient hospital using its cluster’s BPM

Actual inputs and outputs		Beds	Physicians	Nurses	Adjusted Inpatients	Outpatients	Surgeries	Efficiency
		256	46.5	172.92	19,474.2	7,175	220	0.7423
Projections		188 (-27%)	36.9 (-21%)	130.97 (-24%)	19,474.2 (0%)	15,085.3 (110%)	2,170.8 (887%)	1.0000
Improvement scenarios	1	-5%	-10%	-5%	0%	10%	20%	0.7462
	2	-10%	-10%	-5%	0%	10%	40%	0.7526
	3	-15%	-15%	-10%	0%	10%	60%	0.7708
	4	-20%	-15%	-10%	5%	20%	80%	0.7964
	5	-25%	-20%	-10%	5%	20%	100%	0.8907
	6	-30%	-20%	-15%	5%	20%	150%	0.9599
	7	-35%	-10%	-15%	10%	30%	150%	0.9958
	8	-40%	-10%	-15%	10%	30%	150%	1.0250
	9	-45%	-10%	-15%	10%	30%	0%	1.0224
	10	-50%	-10%	-15%	10%	30%	0%	1.0374

Furthermore, we conduct additional experimentation to explore the potential of the proposed framework based on the leader-follower strategy. The results presented in Table 7 can also be utilized to measure hospitals’ efficiency within a managerial network. In cases where a leader-follower strategy can be applied, managers of inefficient or weakly-efficient hospitals can utilize the BPM(s) of their leader(s) as well. Consider a hospital that is part of a private hospital group with 15 hospitals in Cluster 2 and 10 hospitals in Cluster 1, which is the leader of Cluster 2. As reported in Table 11, the relative efficiency score obtained from the SBM DEA model for this hospital is 0.5797 based on original inputs and outputs. The projection of this hospital suggests that drastic changes would be required to become an efficient hospital in its Cluster 2: reducing the number of beds by 33%, physicians by 53%, and nurses by 40%, and increasing the number of outpatients, and surgeries by 2% and 35%, respectively. As a result of Scenario 5, we need less reduction in inputs and less expansion of outputs generated by the hospital to become efficient when using $BP{M}_{1}$ (leader).

**Table 11:** Possible improvement scenarios for another inefficient hospital using its leader’s BPM

Actual inputs and outputs		Beds	Physicians	Nurses	Adjusted Inpatients	Outpatients	Surgeries	Efficiency
		341.0	130.5	275.2	18,313.5	22,221.0	12,969.0	0.5797
Projections		226.8 (-33%)	61.8 (-53%)	165.2 (-40%)	18,313.5 (0%)	22,717.5 (2%)	17,564.8 (35%)	1.0000
Improvement scenarios	1	-5%	-10%	-5%	0%	0%	5%	0.9055
	2	-10%	-10%	-10%	0%	0%	10%	0.9248
	3	-15%	-15%	-15%	0%	2%	15%	0.9531
	4	-20%	-15%	-20%	0%	2%	20%	0.9717
	5	-25%	-20%	-25%	0%	2%	25%	0.9969
	6	-30%	-20%	-30%	0%	5%	30%	1.0159
	7	-35%	-30%	-35%	5%	10%	35%	1.0472
	8	-40%	-30%	-40%	5%	15%	0%	1.0621
	9	-45%	-30%	-45%	5%	0%	0%	1.0677
	10	-50%	-30%	-50%	10%	0%	0%	1.0891

The results show that a nondiscriminatory standard DEA for all hospitals would fail to account for differences in scale heterogeneity, differences in transformational capacities, and likely other exogenous factors that vary between hospitals of the same group. The non-linear mapping and adaptive prediction capabilities of our trained BPMs allow for the compensation of the lack of predictive capabilities of standard DEA models, which are still frequently used as benchmarking tools. Therefore, the framework proposed in this study can assist managers in setting any performance targets for their hospitals over time.

## Conclusions

There are limited economic resources available to hospitals. Therefore, it is essential to determine how the resources are being utilized and whether they are being distributed appropriately. DEA has been used in numerous studies. However, if hospitals operate under different environments, basic DEA alone may not be the best approach and may need some complementary approaches to deal with violations of its assumptions. In this study, we propose a framework for improving the discriminatory and estimation power of DEA. Traditional DEA classifies DMUs in the sample as efficient or inefficient, whereas the proposed framework can account for heterogeneity as a result of the size of the dataset and its ability to transform the data. As complementary to DEA, the framework designs two different architectures of neural networks, namely SOM-ANN and MLP-ANN.

The framework examines the hospital dataset that the Federal Joint Committee of Germany recorded in 2017. To ensure complete accuracy and robustness in calculations, many preprocessing steps are involved in each stage of the framework due to the vast and complex dataset. The proposed framework possesses improved prescriptive capabilities over DEA approaches in a heterogeneous environment. The developed framework may also contribute to the creation of continuous improvement opportunities by promoting the best management practices within a group of hospitals. The proposed framework advances the current benchmarking paradigm of hospitals by learning the optimal performance pattern of hospitals on the efficient frontier of each group. By using what-if and identifying improvement scenarios, the framework can assist decision-makers in evaluating efficiencies. There are clearly defined stages in this study’s framework, and different methods are employed as part of each stage. Analyzers can address the effect of environmental variables on heterogeneity without adding additional variables to DEA models. The key findings of this study can be summarized as follows:

- Natural clustering of hospitals (i.e., based on ownership or size) would not reveal homogeneity within groups of hospitals, nor would it identify heterogeneity between groups of hospitals.
- According to the SBM DEA estimates, the distribution underlying the bootstrapped DEA estimates is identical to the distribution underlying the SBM DEA estimates.
- The differences in the relative efficiency of some German hospitals can be due to differences in their transformation capacities rather than inefficient input usage in producing outputs. Furthermore, a part of the reason for the disparities between the relative efficiency scores of hospitals is scale heterogeneity.
- The trained BPMs can compensate for the lack of predictability of standard DEA models due to their nonlinear mapping and adaptive prediction abilities.

Most studies ignore the heterogeneity pitfall even though it is widely recognized that DEA studies can be compromised by it. DEA would be more robust if methods were developed to prove the reliability and correctness of results. DEA models alone cannot resolve the major problems in hospital performance management that arise from operating in an environment heterogeneous in nature. Because exogenous factors are complex and multiplicative, identifying and measuring them is challenging. Consequently, the process of selecting a reference set for every hospital should be handled cautiously. As demonstrated by well-established quality indicators, it is interesting to note that, contrary to previous findings (Tiemann et al. 2012; Herr 2008), clustering hospitals based on ownership failed to create homogeneity within a group and heterogeneity between groups of hospitals under study. The findings are also different from what one would intuitively expect to find in the context of performance management of hospital markets. For example, one could assume that the relative homogeneity of hospitals would allow for simple emulation of successful policies: if a hospital pursues the goal of increasing its output production efficiency, then such a goal can be accomplished by adopting the strategy of a better-performing peer. However, the adoption of a strategy of a better-performing hospital may not work in the German hospital market since not all hospitals represent a homogenous group. As the results of our clustering show, not every better-performing hospital is a better-performing peer for any other hospital. Nevertheless, we acknowledge this research is not without limitations. While clustering has been used to determine heterogeneity, it remains unclear what exactly constitutes heterogeneity. As heterogeneity is a relative concept that often requires intimate knowledge of the problem domain, this issue falls outside the scope of this study. The proposed framework can therefore be explored further in future research to examine the sources of heterogeneity, such as the differences in hospital environments.

## Figures and Tables

**Fig. 3:**
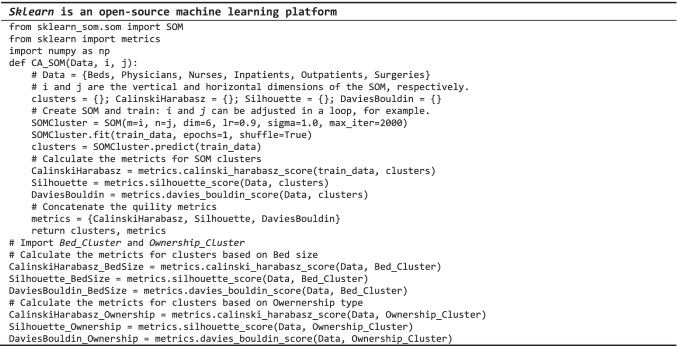
Function developed for clustering based on the SOM-ANN

**Fig. 4:**
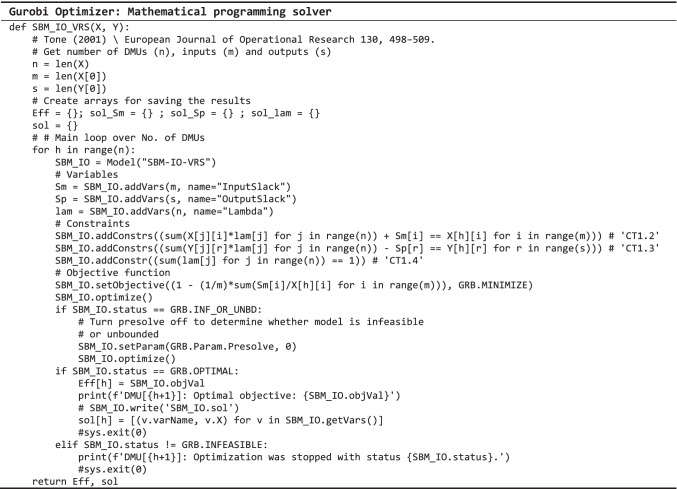
Function developed for solving SBM DEA model

**Fig. 5:**
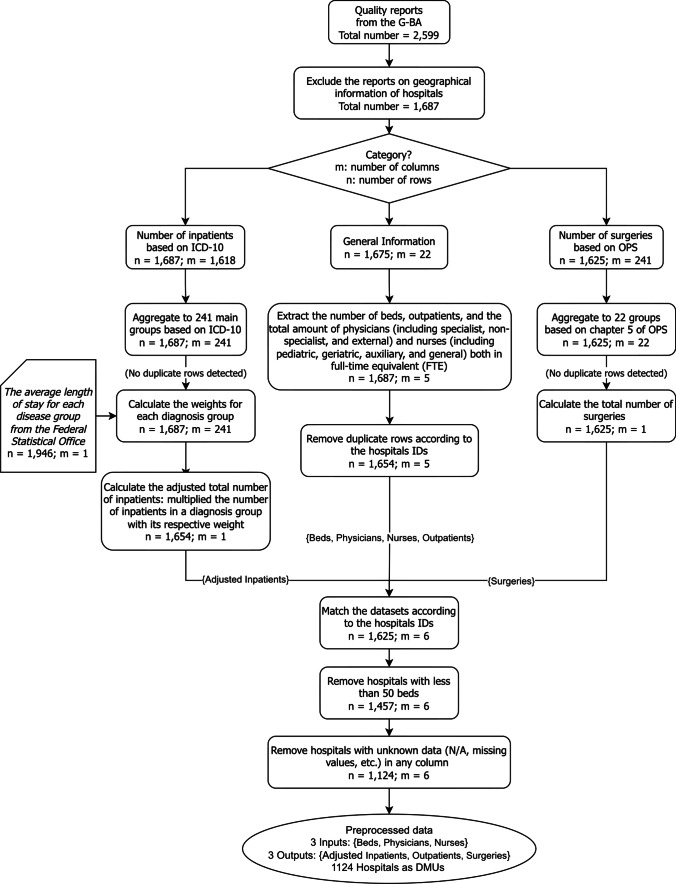
Data preprocessing steps

**Fig. 6:**
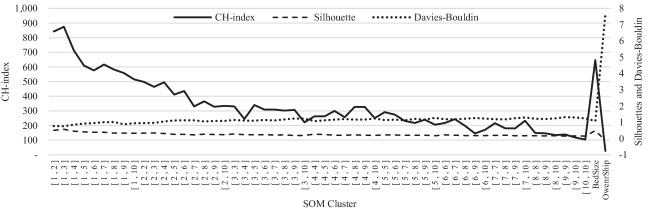
Quality criteria of clusters

**Fig. 7:**
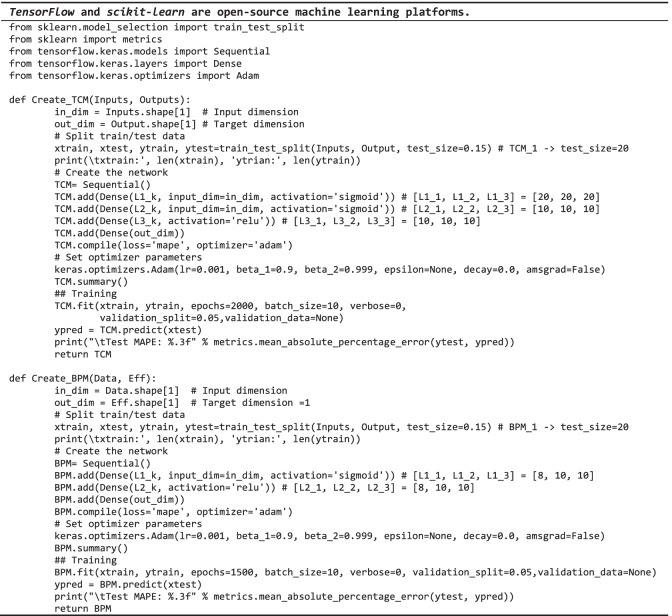
Functions developed for creating TCMs and BPMs

## Appendix A. SOM function

In Figure 3, we present the function developed and used for clustering which is based on the SOM-ANN. The function is developed by using *Scikit-learn* (https://scikit-learn.org/) which is an open-source platform for machine learning. However, the main codes can be also provided upon request.

## Appendix B. Input-oriented SBM DEA model under VRS

We have a set of hospitals in each cluster as ${U}_{j}$ $\forall j\in N=\left\{\mathrm{1,2},\dots ,n\right\}$, each hospital having $m$ inputs ${\varvec{X}}=\left({x}_{1j},{x}_{2j},\dots , {x}_{mj}\right)$ and $s$ outputs ${\varvec{Y}}=\left({y}_{1j},{y}_{2j}\dots , {y}_{rj}\right)$. The linear input-oriented SBM model under the VRS assumption can be written as Model (1).

1.1 $$\mathrm{min}{\rho }_{h}=1-\frac{1}{m}\sum_{i=1}^{m}\frac{{s}_{i}^{-}}{{x}_{ih}}$$

1.2 $$\begin{array}{cc}\mathrm{s}.\mathrm{t}.& {x}_{ih}=\sum_{j=1}^{n}{x}_{ij}{\lambda }_{j}+{s}_{i}^{-}, \forall i=1,\dots ,m\end{array}$$

1.3 $${y}_{rh}=\sum_{j=1}^{n}{y}_{rj}{\lambda }_{j}-{s}_{r}^{+}, \forall r=1,\dots ,s$$

1.4 $$\sum_{j=1}^{n}{\lambda }_{j}=1$$

1.5 $${{\varvec{s}}}^{-},{{\varvec{s}}}^{+},{\varvec{\lambda}}\ge 0,\boldsymbol{ }t>0$$

where ${\rho }_{h}$ is the SBM-efficiency score of $DM{U}_{h}$. ${{\varvec{s}}}^{-}$ and ${{\varvec{s}}}^{+}$ are the vector of input and output slacks, respectively. ${\varvec{\uplambda}}$ is a non-negative vector and can modify the production possibility set by imposing some constraints on it, such as the VRS constraint ${\sum }_{j=1}^{n}{\uplambda }_{j}=1$. The optimal solution of the SBM DEA model can be defined as $\left\{{\rho }_{h}^{*},{{\varvec{\lambda}}}^{*},{{{\varvec{s}}}^{-}}^{*},{{{\varvec{s}}}^{+}}^{*}\right\}$. Figure 4 presents the function developed for solving Model (1) using *Gurobi Optimizer* (more information available at: https://www.gurobi.com/) in Python 3.8.

**Definition 1**. (Projection). *The projection of* $DM{U}_{o}=\left({{\varvec{x}}}_{o},{{\varvec{y}}}_{o}\right)$ *onto the efficient frontiers can be defined by an optimal solution of the input-oriented SBM DEA model as* Eq. (2) (Tone, 2001, 2017)*.*

2 $$\left({{\varvec{x}}}_{o}^{p},{{\varvec{y}}}_{o}^{p}\right)=\left({{\varvec{x}}}_{h}-{{\varvec{s}}}^{-*},{{\varvec{y}}}_{h}+{{\varvec{s}}}^{+*}\right)$$

The projected ${DMU}_{h}^{p}=\left({{\varvec{x}}}_{h}^{p},{{\varvec{y}}}_{h}^{p}\right)$ is SBM-input-efficient (Tone 2001). We use the SBM DEA model to compute efficiency scores for each hospital in the second stage of our proposed framework, relative efficiency analysis. Following this, the framework generates projections of the efficiency requirements for each inefficient hospital to become efficient.

## Appendix C. Efficiency comparison of two hospital groups

The algorithm that is developed for efficiency comparison of two DMU groups (${G}_{1}$ and ${G}_{2}$):

- Calculate the skewness of inefficiencies of both groups.
- If the inefficiencies are not skewed (symmetrically distributed), conduct the efficiency comparison based on the mean values. A parametric test such as the unpaired Student’s t-test might be appropriate (Banker et al. 2010).
- If the inefficiencies are either positively or negatively skewed (asymmetrically distributed), the following are the procedures for testing the null hypothesis of a difference in efficiency between ${G}_{1}$ and ${G}_{2}$:

- Step 3.1: Determine whether inefficiencies in ${G}_{1}$ and ${G}_{2}$ exhibit exponential distributions by using the Quantile-Quantile (Q-Q) plots. If so, the test statistic is therefore calculated as $\left(\sum_{j\in {G}_{1}}{\rho }_{j}^{*}/\Vert {G}_{1}\Vert \right)/\left(\sum_{{j}^{\mathrm{^{\prime}}}\in {G}_{2}}{\rho }_{{j}^{\mathrm{^{\prime}}}}^{*}/\Vert {G}_{2}\Vert \right)$ and assessed to the critical value of the $F$ distribution with $\left(2\cdot \Vert {G}_{1}\Vert ,2\cdot \Vert {G}_{2}\Vert \right)$ degrees of freedom under the null hypothesis that there is no difference between them (Banker 1993).
- Step 3.2: Determine whether inefficiencies in ${G}_{1}$ and ${G}_{2}$ exhibit half-normal distributions by using Q-Q plots. If so, the test statistic is therefore calculated as $\left(\sum_{j\in {G}_{1}}{\left({\rho }_{j}^{*}\right)}^{2}/\Vert {G}_{1}\Vert \right)/\left(\sum_{{j}^{\mathrm{^{\prime}}}\in {G}_{2}}{\left({\rho }_{{j}^{\mathrm{^{\prime}}}}^{*}\right)}^{2}/\Vert {G}_{2}\Vert \right)$ and assessed to the critical value of the $F$ distribution with $\left(\Vert {G}_{1}\Vert ,\Vert {G}_{2}\Vert \right)$ degrees of freedom under the null hypothesis that there is no difference between them.
- Step 3.3: In the absence of such assumptions in steps 3.1 and 3.2, use a non-parametric test, such as Kolmogorov–Smirnov or Mann–Whitney tests. The results of the study conducted by Banker et al. (2010) indicate that the Mann–Whitney test performs better than Kolmogorov–Smirnov concerning error types I and II. Next, run the Mann–Whitney test to determine whether one of the random variables is stochastically greater than the other. In a combined and ordered sample of ${G}_{1}$ and ${G}_{2}$, the Mann–Whitney statistic is calculated by counting how many times each ${\rho }_{j}^{*}, j\in {G}_{1}$ occurs before ${\rho }_{{j}^{\mathrm{^{\prime}}}}^{*}, {j}^{\mathrm{^{\prime}}}\in {G}_{2}$. Define the random variable as Eq. (3).
  3 $${D}_{j{j}^{\mathrm{^{\prime}}}}=\left\{\begin{array}{c}1 {\rho }_{j}^{*}<{\rho }_{{j}^{\mathrm{^{\prime}}}}^{*} \\ 0\ otherwise\end{array}\right.$$
- Then, Mann–Whitney’s statistic is given by $U=\sum_{j\in {G}_{1}}{\sum }_{{j}^{\mathrm{^{\prime}}}\in {G}_{2}}{D}_{j{j}^{\mathrm{^{\prime}}}}$. Mann and Whitney (1947) prove that for large samples of ${G}_{1}$ and ${G}_{2}$ ($\Vert {G}_{1}\Vert and \Vert {G}_{2}\Vert \ge 30$), Mann–Whitney’s statistic is normally distributed with the mean of $\mu =\Vert {G}_{1}\Vert \cdot \Vert {G}_{2}\Vert /2$ and the variance of ${\sigma }^{2}=\left(\Vert {G}_{1}\Vert \cdot \Vert {G}_{2}\Vert \cdot \left(\Vert {G}_{1}\Vert +\Vert {G}_{2}\Vert +1\right)\right)/12$. Therefore, the large-sample (more than 20 observations) Mann–Whitney’s test statistics can be approximated via $\mathcal{z}=\left(U-\mu \right)/\sigma$ which follows a normal standard distribution function. Note, since there are a number of ties (i.e., the ranks of efficient DMUs) in each cluster, we need to revise the variance as ${\sigma }_{revised}^{2}={\sigma }^{2}\cdot \left(1-\sum_{t}{f}_{t}^{3}-{f}_{t}/{f}_{t}^{3}-{f}_{t}\right)$, where t varies over the set of tied ranks and ${f}_{t}$ represents frequency of the rank $t$. A further complication is that since we approximate a discrete distribution via a continuous one it is desirable to apply a continuity correction on the $\mathcal{z}$-score as ${\mathcal{z}}_{corrected}=U-\mu -0.5\cdot sign\left(U-\mu \right)/\sigma$.

## Appendix D. Data preprocessing

In this study, the proposed framework is examined in the context of a large dataset of hospitals that were originally classified by the Federal Joint Committee (G-BA) in Germany in 2017. Data protection regulations prevent the dataset from being publicly available. Nevertheless, G-BA would send a copy to researchers upon official request (more information: https://www.g-ba.de/english/). In the German healthcare system, the G-BA, founded on 01.01.2004 due to the Healthcare Modernization Act, is the highest decision-making body. They establish guidelines that determine which medical treatments approximately 73 million insured people can claim. Furthermore, the G-BA establishes quality assurance measures for hospitals and healthcare practices. It is their responsibility to properly implement quality-improving measures. The implementation of individual quality assurance measures should be delegated as part of this overall responsibility. For the reporting year 2017, raw data includes all hospital quality reports from hospitals, the State Office for Quality Assurance, and the Institute for Quality Assurance and Transparency in Health Care at the end of medical transcription (MT). The preprocessing steps applied to the dataset in this study are illustrated in Figure 5. Our dataset covers the following periods:

- Hospitals MT periods: October 15th to November 15th, 2018, and November 23rd to December 15th, 2018,
- State Office for Quality Assurance and Institute for Quality Assurance and Transparency in Health Care MT periods: November 15th to December 15th, 2018, and
- the subsequent reports of the State Office for Quality Assurance and the Institute for Quality Assurance and Transparency in Health Care occurring from January 20th to 23rd, 2019.

## Appendix E. Quality criteria for clustering approaches

Figure 6 shows the three quality criteria - CH-index, Silhouettes, and Davies-Bouldin - calculated to assess the homogeneity within hospitals clusters and the heterogeneity between clusters. These criteria are calculated in the function developed for the SOM (Figure 3). Clusters that are dense and well separated achieve a high score on the CH-index. A clustering score of $-1$ is assigned for incorrect clustering, whereas a clustering score of $+1$ is assigned to dense and well-separated clustering. Davies-Bouldin with a value close to zero indicates a more effective partition. Results show that cluster [1,3] outperforms other clusters on all three quality criteria.

## Appendix F. Developed MLP-ANNs for creating TCM and BPM

As shown in Figure 7, we have developed functions to create the TCMs and BPMs respectively by using two open-source platforms for machine learning: *TensorFlow* (more information available at: https://www.tensorflow.org/) and *Scikit-learn* (more information available at: https://scikit-learn.org/).

## Footnotes

1 Press Release No. 435 as of November 12, 2018

2 In German: Gemeinsamer Bundesausschuss. https://www.g-ba.de/

3 https://www.destatis.de/

4 Chapter 5 of OPS (Operationen- und Prozedurenschlüssel) which is the German modification of the International Classification of Procedures in Medicine.
